# The role of peroxidasin in solid cancer progression

**DOI:** 10.1042/BST20230018

**Published:** 2023-10-06

**Authors:** Kaitlin Wyllie, Vasilios Panagopoulos, Thomas R. Cox

**Affiliations:** 1 Matrix & Metastasis Lab, The Garvan Institute of Medical Research & the Kinghorn Cancer Centre, Cancer Ecosystems Program, Sydney, NSW 2010, Australia; 2 School of Clinical Medicine, St Vincent's Healthcare Clinical Campus, Faculty of Medicine and Health, UNSW Sydney, Sydney, NSW, Australia; 3 Myeloma Research Laboratory, Faculty of Health and Medical Sciences, School of Biomedicine, University of Adelaide, Adelaide, Australia; 4 Precision Cancer Medicine Theme, Solid Tumour Program, South Australian Health and Medical Research Institute, Adelaide, Australia

**Keywords:** cancer, metastasis, peroxidases

## Abstract

Peroxidasin is a heme-containing peroxidase enzyme that plays a vital role in the cross-linking of collagen IV molecules in basement membranes. Collagen IV cross-links are essential for providing structure and mechanical stability throughout tissue development, homeostasis, and wound healing. During cancer progression, the basement membrane is degraded, and proteins typically found in the basement membrane, including peroxidasin and collagen IV, can be found spread throughout the tumour microenvironment where they interact with cancer cells and alter cell behaviour. Whilst peroxidasin is reported to be up-regulated in a number of different cancers, the role that it plays in disease progression and metastasis has only recently begun to be studied. This review highlights the current literature exploring the known roles of peroxidasin in normal tissues and cancer progression, regulators of peroxidasin expression, and the reported relationships between peroxidasin expression and patient outcome in cancer.

## Introduction

**Figure 1.**
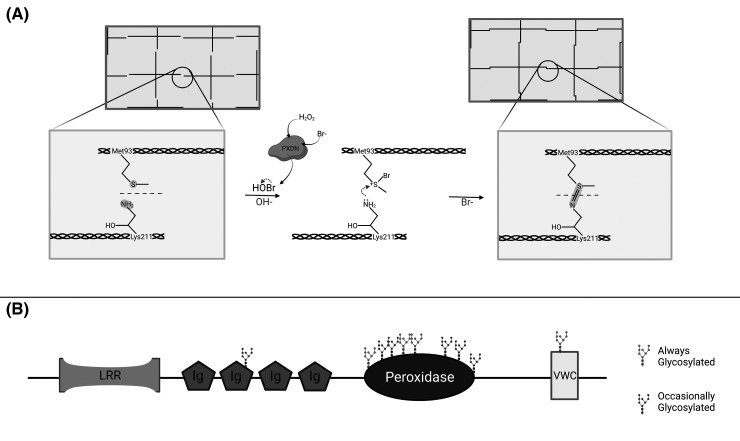
Activity and protein structure of PXDN. (**A**) HOBr generated by PXDN facilitates formation of sulfilimine cross-links between Met93 and Lys211 on collagen IV molecules. (**B**) Human PXDN contains an LLR domain followed by four repeated Ig domains, a peroxidase domain and the C-terminal VWC domain. There are four sites that have been reported to be always glycosylated, and six additional glycosylation sites that are glycosylated part of the time. Created with BioRender.com.

The basement membrane is a thin layer of highly specialised extracellular matrix that underlies epithelial and endothelial cells. It plays many important roles including providing structural support, determining the polarity of anchored cells, regulating cell growth and differentiation, and acting as a filter to regulate the movement of molecules between the bloodstream and the tissue [1,2]. During injury and tissue repair, the basement membrane also facilitates aligning cells for migration and re-epithelialization [1]. Collagen IV is the most abundant component present and plays an important role in providing structure to the basement membrane. To stabilise the collagen IV network, collagen IV trimers are cross-linked via their NC1 domains through sulfilimine bond formation between the sulfur group of Met93 and the amine nitrogen on hydroxy-Lys211 (Figure 1A) [3]. This cross-linking is mediated by the potent oxidant hypobromous acid (HOBr), generated by the peroxidase enzyme, peroxidasin (PXDN), and relies on the presence of both hydrogen peroxide and bromide [4,5]. This PXDN-catalysed sulfilimine bond formation is an evolutionarily conserved structural feature of collagen IV networks that is known to be essential for tissue development and function [6].

PXDN is a heme-containing enzyme first identified in the cardiovascular system [7]. Other members of the mammalian heme peroxidase family include myeloperoxidase (MPO), eosinophil peroxidase (EPX or EPO) and lactoperoxidase (LPO). Peroxidases utilise hydrogen peroxide to form highly reactive intermediates, which then oxidise halide ions (such as bromide, chloride, and thiocyanate) to form hypohalous acids. Both MPO and EPO are key elements of the innate immune response, while LPO is found in exocrine secretions. The hypohalous acids generated by these enzymes are well-characterised for their antimicrobial properties. PXDN, however, has a unique function in the cross-linking of collagen IV molecules. MPO oxidises Cl^−^ to generate hypochlorous acid (HOCl), LPO oxidises SCN^-^ to generate hypothiocyanous acid (HOSCN), whereas, both PXDN and EPO utilise Br^-^ ions to form hypobromous acid (HOBr) [8–11]. Whilst there is some evidence to suggest that PXDN may have some limited capacity to form HOCl in addition to HOBr [9], this activity is thought to be negligible [8,10].

Importantly, although both PXDN and EPO produce HOBr and both have been shown to promote angiogenesis [12,13], only HOBr generated by PXDN appears efficient in forming the sulfilimine cross-links required to stabilise collagen IV networks [9]. Whilst HOBr from EPO is thought to be able to cross-link some proteins, this is much less efficient than PXDN-mediated generation of sulfilimine bonds within the NC1 domains of collagen IV [14]. Additionally, in *in vitro* studies in which cells were transfected with PXDN, MPO and LPO cDNA and seeded onto uncross-linked basement membrane, only cells transfected with PXDN cDNA were able to generate collagen IV cross-links [9]. More recently, lysyl oxidase like-2 (LOXL-2), an amine oxidase, has been shown to promote the formation of lysyl-derived cross-links in the 7S dodecamer, but not the NC1 domain of collagen IV [6]. Thus, to date, cross-linking of the collagen IV NC1 hexameric junctions appears to be exclusively mediated by PXDN.

In this mini-review, we discuss the known and emerging roles of PXDN in cancer progression, the known associations between PXDN expression and patient outcome, and examine the literature surrounding PXDN regulatory pathways and the implications of these in cancer progression.

### PXDN structure and activity

The PXDN enzyme contains four types of domains; the leucine-rich repeat (LLR), immunoglobulin (Ig), peroxidase and von Willebrand factor type C (VWC) domains [8,14–17] (Figure 1B). Both peroxidase and Ig domains are required for optimal sulfilimine bond formation, although studies have shown that bond formation can still occur with the peroxidase domain alone [14]. PXDN is expressed as a monomer, which then assembles into dimers and ultimately a mature homotrimer conformation that is secreted from the cell. These trimers are formed through disulfide bonds, likely between cysteine residues C736 and C1315 [18]. The PXDN protein is also highly glycosylated and has been shown to contain four glycosylation sites and six additional sites that can be glycosylated some of the time (Figure 1B), with glycosylation forming up to 12 kDa of the final mass [8].

The active site required for catalysis of HOBr is in the peroxidase domain which requires heme incorporation for correct function [8]. Association with collagen IV appears to occur through the Ig domains, and is transient [14,15], since a direct binding of PXDN to collagen IV has so far not been shown. However, PXDN has also been shown to be able to interact with laminins through interactions with the leucine-rich repeat domain [15]. This likely facilitates the interaction with collagen IV given their intimately linked roles within the basement membrane, however the exact physiological role for this PXDN-laminin interaction has yet to be determined. Nonetheless, silencing of PXDN expression results not only in a decrease in collagen IV cross-linking, but subsequently reduces laminin and fibronectin content of the basement membrane. Whilst there is no direct evidence to suggest that PXDN cross-links laminin and fibronectin, the close interaction of these with collagen IV suggests that loss of collagen IV network stability has knock-on effects on the biochemistry of the basement membrane.

## The emerging role of PXDN in solid cancers

### Changes in PXDN expression in cancer

**Figure 2.**
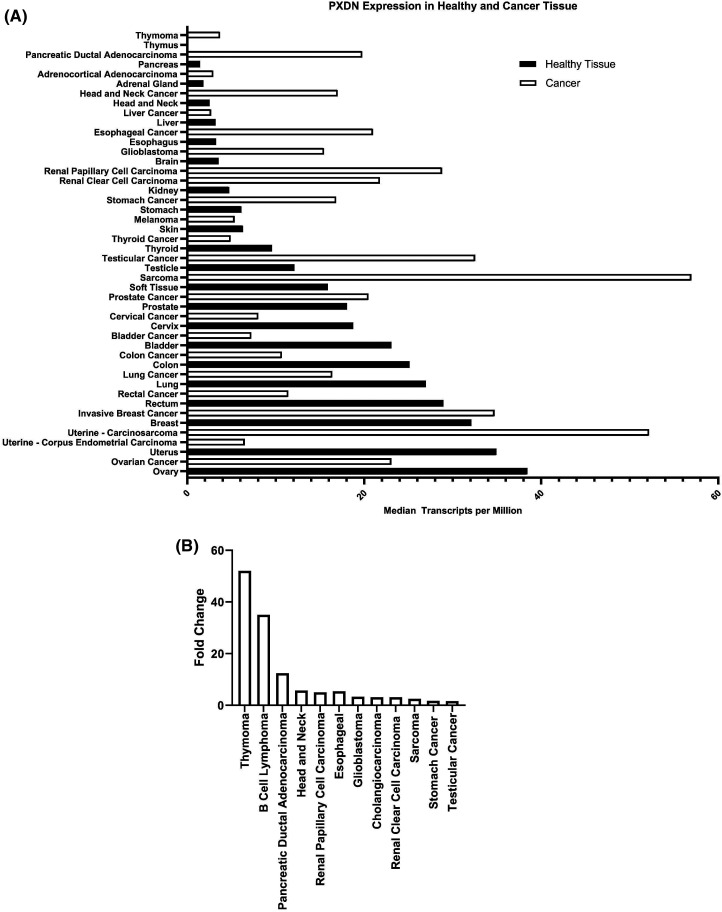
PXDN expression levels in healthy and tumour tissue. Graphs show RNA sequencing data of healthy tissue and corresponding tumour tissue according to data from TCGA and GTEx (collected from GEPIA [39]). (**A**) Median PXDN transcripts per million measured by RNA sequencing. (**B**) Top 12 cancers with the largest fold change in PXDN expression levels in tumour tissue compared with healthy tissue.

**Figure 3.**
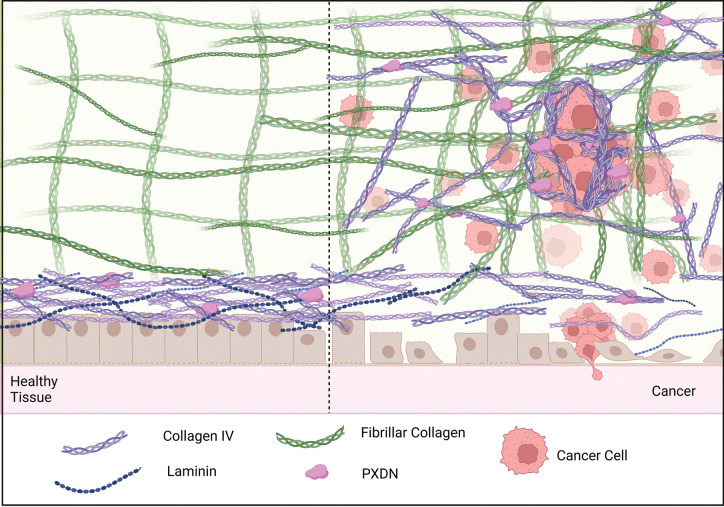
Changes in basement membrane and ECM during cancer progression. In solid tumours, there is a loss of basement membrane integrity and structure facilitating increased cell invasion into the vasculature and lymphatic systems. Simultaneously, the ECM of the tumour microenvironment becomes dysregulated with increased and aberrant deposition of fibrillar collagens as well as network forming collagens such as collagen IV. PXDN levels are also often elevated within solid tumours, and in light of the known collagen IV cross-linking function of PXDN, this may lead to stablisation of aberrant collagen IV networks within the tumour microenvironment. Created with BioRender.com.

Compared with matched healthy tissue, PXDN is up-regulated in a number of different cancers (Table 1 and Figure 2) including melanoma [19–21], sarcoma [22], glioblastoma [22–24], oral carcinoma [25,26], prostate and testicular cancer [22,27], ovarian cancer [22,28], pancreatic cancer [22,29], thyroid cancer [30], head and neck cancer [22] and stomach cancer [22]. In these studies, PXDN expression has been measured by multiple approaches including mass spectrometry, RT-PCR and/or IHC. Typically PXDN has been shown to increase with advancing stages of disease, such as in prostate [27], testicular [22], colon [22], bladder [22,31], and ovarian cancer [32] (Figure 3). High expression of PXDN has also been reported to be associated with reduced overall survival in ovarian [28], bladder [31], gastric [33,34] and uterine [35] cancers as well as sarcomas [36].

**Table 1:** Expression and function of PXDN in cancer

Cancer type	Expression of PXDN^1^	Reported phenotype	Techniques used	Refs.
Melanoma	Up-regulated	High levels of PXDN increase EMT in melanoma cells.	Comparison of PXDN expression levels in a range of epithelial-like and mesenchymal-like cell lines.	[19,22]
Glioblastoma	Up-regulated	Bioinformatic pan-cancer analysis associating PXDN with patient outcome	Systematic pan-cancer analysis of PXDN using data from TCGA, GTEx, and related databases to compare PXDN expression with clinical characteristics.	[22–24]
Oral carcinomas	Up-regulated	Patients with high PXDN levels had worse disease free survival, lower levels of Reactive Oxygen Species (ROS), increased ATP production and increased lymph node metastasis.	Bioinformatic analysis examining the association between PXDN expression and patient outcomes.	[25,26]
Prostate cancer	Up-regulated	Conditioned media was able to induce migration. Knockdown of PXDN resulted in increased cellular ROS and decreased cell viability and tumorigenicity.	Treatment of cells with conditioned media containing PXDN. shRNA knockdown of PXDN in C4-2 prostate cancer cells	[22,27,65]
Testicular cancer	Up-regulated	Bioinformatic pan-cancer analysis associating PXDN with patient outcome	Systematic pan-cancer analysis of PXDN using data from TCGA, GTEx, and related databases to compare PXDN expression with clinical characteristics.	[22]
Ovarian cancer	Up-regulated	PXDN knockdown reduced proliferation, migration and invasion of cancer cells, as well as reduced PI3K and AKT phosphorylation.	siRNA knockdown of PXDN in HEY ovarian cancer cells.	[22,28]
Pancreatic cancer	Up-regulated	Bioinformatic pan-cancer analysis associating PXDN with patient outcome	Systematic pan-cancer analysis of PXDN using data from TCGA, GTEx, and related databases to compare PXDN expression with clinical characteristics.	[22,29]
Thyroid cancer	Up-regulated	PXDN is part of a four gene signature that showed diagnostic potential in anaplastic thyroid cancer for molecular targeted therapy and immunotherapy.	Exploratory study using ATC RNA-sequencing data from the GEO database, linking PXDN expression to immune infiltration	[30]
Head and neck cancer	Up-regulated	Bioinformatic pan-cancer analysis associating PXDN with patient outcome	Systematic pan-cancer analysis of PXDN using data from TCGA, GTEx, and related databases to compare PXDN expression with clinical characteristics.	[22]
Stomach cancer	Up-regulated	Bioinformatic pan-cancer analysis associating PXDN with patient outcome	Systematic pan-cancer analysis of PXDN using data from TCGA, GTEx, and related databases to compare PXDN expression with clinical characteristics.	[22]
Liver (Hepatocellular Carcinoma)	Up-regulated	Bioinformatic pan-cancer analysis associating PXDN with patient outcome	Systematic pan-cancer analysis of PXDN using data from TCGA, GTEx, and related databases to compare PXDN expression with clinical characteristics.	[22]
Uterine serous carcinoma	Up-regulated	Bioinformatic pan-cancer analysis associating PXDN with patient outcome	Systematic pan-cancer analysis of PXDN using data from TCGA, GTEx, and related databases to compare PXDN expression with clinical characteristics.	[22]
Bladder cancer	Down-regulated	Bioinformatic pan-cancer analysis associating PXDN with patient outcome	Systematic pan-cancer analysis of PXDN using data from TCGA, GTEx, and related databases to compare PXDN expression with clinical characteristics.	[22]
Uterine corpus endometrial carcinoma	Down-regulated	Bioinformatic pan-cancer analysis associating PXDN with patient outcome	Systematic pan-cancer analysis of PXDN using data from TCGA, GTEx, and related databases to compare PXDN expression with clinical characteristics.	[22]
Breast cancer	No significant change	Conditioned media was able to induce migration. Knockdown of PXDN resulted in decreased cell proliferation. Pateints with high PXDN levels had poorer metastasis free survival	Treatment of cells with conditioned media containing PXDN. siRNA knockdown of PXDN in MCF10A cells. Bioinformatic analysis examining the association bewteen PXDN expression and pateint outcomes.	[22,37,65,76]
Colon cancer	No significant change	Bioinformatic pan-cancer analysis associating PXDN with patient outcome	Systematic pan-cancer analysis of PXDN using data from TCGA, GTEx, and related databases to compare PXDN expression with clinical characteristics.	[22]
Lung carcinoma	N/A	Fine particulate matter in air pollution activates PXDN to increase collagen IV cross-linking and decreased cytotoxic T lymphocyte migration in lung cancer	Intra-tracheal delivery of shRNA targeting PXDN into the lungs of mice.	[66]

1 Expression in comparison with healthy tissue of the same tissue type.

Interestingly, no association has been seen between overall survival and PXDN expression levels in oral squamous cell carcinoma and breast cancer, however in both of these cancers there is reported to be an association between high PXDN expression and poorer disease-free or metastasis-free survival [25,37]. As such, these data suggest a possible role for PXDN in also mediating metastatic dissemination and relapse. There is some evidence to suggest that PXDN expression is highest in mid-stage disease in breast cancer, and begins to decrease again at later stages of disease [38], although what this might mean biologically is unknown. Additional studies stratifying patient outcomes and expression levels of PXDN according to disease stage and/or aggressiveness would help to begin to better understand these differences.

### Cell types expressing PXDN in solid cancers

There are limited studies examining the source of expression of PXDN in cancer. PXDN is predominantly expressed by epithelial and endothelial cells in healthy tissue where they are responsible for lens formation in the eye and vascular basement membrane deposition, respectively [16,40,41]. However, PXDN has also been shown to be highly expressed by cancer cells in various cancers [25,27,28,41–45], as well as by some fibroblasts [37] especially during tissue fibrosis [46]. Thus, it is likely that PXDN may be expressed by cancer-associated fibroblasts (CAFs) especially in tumours with a high degree of desmoplasia.

PXDN is highly expressed in cancer cells undergoing epithelial to mesenchymal transition (EMT), a process which underpins metastatic dissemination and poor patient outcomes [47,48]. Whether elevated PXDN expression drives EMT, or is simply a result of EMT, remains to be seen. In a range of melanoma cell lines of varying aggressiveness, PXDN expression was higher in more mesenchymal-like cells [19], which has also been observed in some breast cancer models [37,49]. Therefore, PXDN may be expressed in tumour regions undergoing EMT although the biological mechanisms underpinning this remain to be elucidated. In future, spatial proteomics studies would be highly beneficial in determining the spatial location of PXDN within tumours.

### PXDN and collagen IV cross-linking in the tumour microenvironment

The primary known role of PXDN is cross-linking of collagen IV. This is required to provide structural integrity to the basement membrane in normal tissue maintenance and during repair. In cancer, there is commonly a loss of basement membrane integrity. The loss of the basement membrane structure as well as alterations in its biomechanical properties subsequently provides opportunity for increased cell invasion through the basement membrane, a key step associated with metastatic dissemination [50–52]. Collagen IV also becomes aberrantly deposited within the tumour microenvironment, where it interacts with cancer cells and other extracellular matrix molecules to promote disease progression [50–53]. For example, collagen IV is deposited around pancreatic cancer cells encapsulating them within a pseudo-basement membrane-like structure [54,55] (Figure 3). The arrangement of collagen IV may also directly contribute to tumour progression and patient outcome, since thick filament bundle of collagen IV around clusters of cells is observed in well-differentiated cancers, which typically have poor prognosis, whilst non-differentiated models exhibit an arrangement of collagen IV with thinner fibres around individual tumour cells [55].

### Potential alternative roles for PXDN and their implications in cancer

PXDN effects on cells are most likely through its collagen IV cross-linking activity, yet there is evidence to suggest that PXDN may have additional functions relevant to the cancer setting. For example, the capacity of the LRR domain in PXDN to interact with laminin [15] has raised questions about a possible role for PXDN in the oxidative modification of laminin and other nearby ECM components, especially given that HOBr is known to cross-link proteins. PXDN may also play a role in heme oxygenase-1 (HO-1) mediated adhesion of BeWo LMP cells to laminin and fibronectin matrices [45], suggesting that PXDN may have the capacity to influence adhesion to other ECM components, however, it is not clear what the mechanism responsible for this is.

Alternately, some studies have hypothesised that PXDN's use of hydrogen peroxide to catalyse the formation of HOBr, both of which are forms of reactive oxygen species (ROS), may be altering the levels of oxidative stress experienced by cancer cells. In non-cancerous aortic and endothelial cells, knockdown of PXDN with siRNA or shRNA led to a decrease in superoxide and ROS levels during oxidative stress [41,56]. In prostate cancer, however, the opposite trend was observed, with PXDN depletion in C4-2 cancer cells resulting in an increase in cellular ROS [27]. Furthermore, in oral squamous cell carcinoma, ROS production was found to have an inverse relationship with PXDN expression [25].

PXDN driven changes in oxidative stress may also be mediated through alterations in cellular energetics and metabolism. Purine metabolism is known to be increased in cells to mitigate oxidative stress [57]. Uric acid, an end product of purine metabolism, has been reported to be a possible inhibitor of PXDN [58,59]. Moreover, high levels of PXDN have also been associated with increased ATP production [25], a typical hallmark of cancer metabolism, however there is to our knowledge no direct link between ATP production and PXDN activity so it is likely that there may be confounding variables which were not identified in this study.

Treatment of cells with excess exogenous fatty acids, such as palmitate, is known to trigger apoptotic cell death through impairment of autophagic flux. In cardiomyocytes treated with palmitate, increased levels of PXDN were observed, as well as reduced glucose consumption and impairment of autophagic flux, resulting in higher oxidative stress and subsequent cell death [60]. siRNA depletion of PXDN in these cells improved autophagic flux and consequently reduced cell death. Given that cellular energetics are often altered in cancer cells, future studies could examine the link between glucose metabolism, autophagic flux, oxidative stress and PXDN in the cancer setting.

## Mechanisms of regulation of PXDN expression and activity

### Regulation of PXDN during normal tissue homeostasis

**Figure 4.**
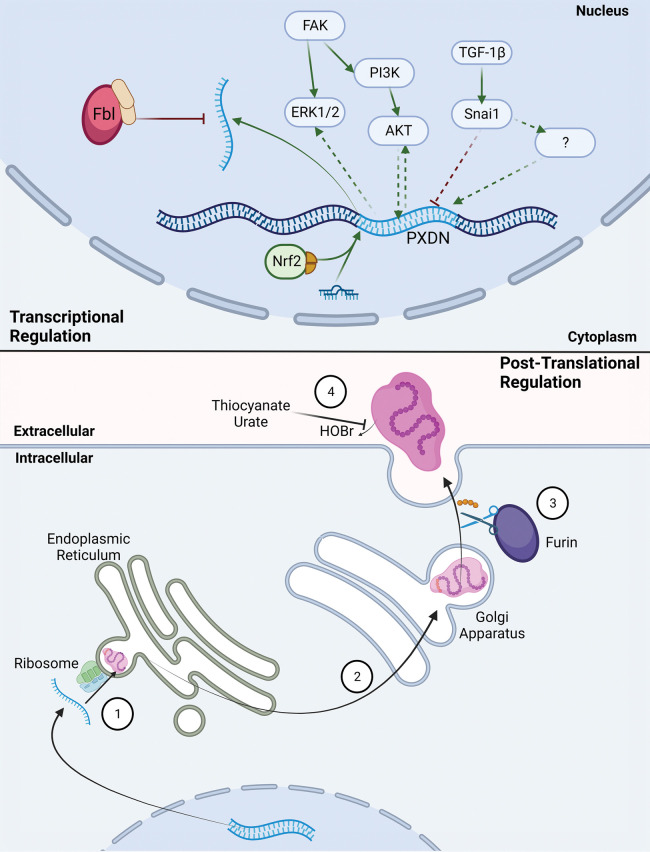
Regulation of PXDN at the transcriptional and post-translational level. At the transcriptional level, PXDN expression can be increased by binding of nuclear factor Nrf2 when joined with inducers tBHQ and SFN, or microRNAs miR-203 or miR-29b-3p, to the promoter region of the PXDN gene. The Snai1, FAK/PI3K/AKT and FAK/ERK signalling pathways have all been linked to regulation of PXDN expression, although the exact mechanisms underlying this regulation have yet to be properly understood (dotted lines). The 2′-O-methylase Fbl, guided by snorU32A and U52, can methylate PXDN mRNA to reduce levels of transcription. Once PXDN mRNA exits the nucleus, it is co-translationally inserted into the ER as full length, immature protein (1), before being transported through the endoplasmic reticulum and golgi apparatus via the classical secretory pathway (2) and secreted from the cell. Prior to secretion, the c-terminal peptide is cleaved from the full length PXDN protein by the pro-protein convertase Furin to form the mature PXDN protein (3). Activity of the mature PXDN protein can be moderated by thiocyanate and urate, which act as alternative substrates for PXDN, resulting in reduced HOBr production and therefore reduced cross-linking activity (4). Created with BioRender.com.

Due to the critical role of the basement membrane in tissues and the importance of correctly cross-linked collagen IV, the regulation of PXDN is key to basement membrane integrity. The exact mechanisms and pathways that regulate PXDN expression are yet to be fully understood. At the post-transcriptional level, the 2′-*O*-methylase *Fbl* has been shown to modify PXDN mRNA when guided by small nucleolar RNAs U32A and U51 (Figure 4), which both have high complementarity to a conserved coding region of PXDN mRNA [61]. mRNA methylation has also been shown to decrease translation and production of PXDN [61]. In other work, the microRNA miR-29b-3p, which is known to be up-regulated during cell stress, has been shown to increase PXDN expression levels in cardiac cells *in vitro* [62].

The immature PXDN protein is post-translationally modified to form mature PXDN before secretion from the cell (Figure 4). The C-terminus of PXDN is cleaved at arginine 1336 by the pro-protein convertase furin, resulting in the removal of a 30 kDa propeptide [63,64]. Full length PXDN has been shown to be able to generate HOBr and cross-link collagen IV when added exogenously to cultures *in vitro*, however this activity is significantly higher in the mature, processed form of PXDN [63,64]. Whilst full length PXDN can be detected both intracellularly and extracellularly, only cleaved PXDN is located extracellularly suggesting that proteolytic cleavage occurs at a late stage, just prior to secretion [63]. In support of this, addition of an ER retention signal (KDEL) to the C-terminus of PXDN significantly reduced its secretion by PFHR-9 cells, in turn reducing collagen IV cross-linking [63].

Regulators of PXDN enzymatic activity are not well understood, however physiological levels of thiocyanate and urate have been shown to partially inhibit PXDN cross-linking activity [5]. Given that urate is an end product in purine metabolism (discussed above), and this metabolic pathway is increased in cells undergoing oxidative stress, it is possible that urate may regulate PXDN during oxidative stress [57].

### Effects of PXDN dysregulation in tumour progression

The exact role that PXDN plays in the progression of different cancers remains unclear and is likely multifaceted. Abnormal expression of PXDN in tumours has been linked to changes in cell viability, proliferation, migration, and invasion. Elevated PXDN was among the main ECM effectors identified in conditioned media from bone marrow derived mesenchymal stromal cells that induced migration of both PC3 prostate and MDA-MB-231 breast cancer cells [65]. Short hairpin RNA (shRNA) depletion of PXDN in C4-2 prostate cancer cells also reduced cell viability and colony forming ability in soft agar [27]. In ovarian cancer patient tumours, high expression of PXDN is associated with smaller tumours [32], yet *in vitro* depletion of PXDN in HEY human ovarian cancer cells reduced proliferation and migration [28]. Increased levels of PXDN in oral squamous cell carcinoma have also been associated with increased lymph-node metastasis and infiltration at the primary site [25], and high expression of PXDN is associated with shorter metastasis-free survival in breast cancer [37]. These data highlight how the tumour type, source of PXDN (autocrine vs. paracrine), setting (*in vitro* vs. *in vivo* vs. patient) as well as experimental setup may yield contrasting results.

PXDN mediated cross-linking of collagen IV has been linked to changes in immune infiltration, which may play an important role in solid tumour progression. Intra-tracheal delivery of PXDN shRNA into the lungs of mice reduced collagen IV cross-linking and subsequently increased cytotoxic T lymphocyte migration into the lungs [66]. These data strongly suggest that PXDN and/or collagen IV cross-linking may be important in altering the tumour microenvironment and therefore immune cell infiltration in some solid cancers, although this has yet to be tested formally.

Expression of PXDN has been linked to vascular tube formation and angiogenesis in endothelial cells [12,13]. Angiogenesis is crucial in solid tumours as it enables the formation of new blood vessels, providing tumours with nutrients and oxygen, fuelling their growth and metastasis. However, a role for PXDN in tumour angiogenesis has yet to be investigated.

### Transcriptional (Dys)-regulation of PXDN expression in cancer

A range of transcriptional regulatory pathways of PXDN expression have been identified in different cancer cells, including methylation, transcription factors and microRNAs (Figure 4). Methylation of the promoter region of PXDN appears to suppress transcription and is negatively correlated with PXDN expression in cancers of the liver, breast, skin, kidney, oesophagus, thyroid, rectum, lung, bladder, stomach, bone, head and neck, colon, prostate, mesothelium, pancreas, uterus, cervix and ovary [22]. In prostate cancer, PXDN promoter methylation has been shown to be mediated through PRMT5 leading to an increase in expression of PXDN [67]. Contrary to these results, methylation frequency of PXDN appears to increase with disease progression in pancreatic cancer [68], whilst methylation status of PXDN showed no significant association with patient outcomes in colorectal cancer [69].

PXDN typically exhibits a lower mutational burden than many common oncogenes. However, mutations in the PXDN gene appear frequently in uterine cancers [22], stomach cancer [22], melanoma [21,22] and colon cancer [22,70]. The most common type of mutations of the PXDN gene in cancers are missense mutations [21,22,36,70], however the pathology of many of these, including potential effects on enzymatic activity, if any, remains unknown. Survival analyses performed in cohorts of melanoma patients found no direct association between mutations in the PXDN gene and overall survival [21], yet Paumann-Page et al. [20] recently demonstrated a correlation of PXDN expression and survival in melanoma. However, with limited or no functional validation to date, it is not clear whether various mutations in the PXDN gene would alter expression or activity.

The nuclear factor Nrf2 has been shown to increase PXDN expression by HeLa cells through binding to the PXDN promoter region [71]. The Nrf2-mediated oxidative response pathway was also among the most differentially regulated between PXDN knockdown and controls in C4-2 prostate cancer cells [27]. Additionally, a binding site for the microRNA miR-203a has been identified in the 3′ UTR of PXDN [72] suggesting that PXDN may fall under miR regulation, and supporting work has shown that addition or removal of miR-203a was able to increase or decrease expression of PXDN respectively. Complementing this, PXDN has also been predicted to be a target gene of miR-203 in pancreatic cancer [73].

PXDN regulation in cancer may also be a result of dysregulation of Snai1, a transcriptional repressor. ChiP-PCR analysis and functional assays in HeLa and SiHa cells showed binding of Snai1 to the PXDN promoter region upon TGF-b1 stimulation [43] led to repression of PXDN expression. In contrast, in a panel of prostate cancer cell lines, PXDN protein levels were found to be higher in cells expressing Snai1 and vimentin [27]. Additionally, in ARCaP prostate cancer cells transfected with Snai1 cDNA to overexpress Snai1, PXDN expression was significantly fold up-regulated compared with control [42].

### Post-translational (Dys)-regulation of PXDN expression in cancer

The exact regulatory mechanisms of PXDN during homeostasis are not yet fully understood. For this reason, further research into this area in cancer, particularly in comparison with healthy tissue, is needed. A better understanding of the cancer-specific alterations in PXDN expression and activity, the downstream effects of collagen IV cross-linking, and role this plays in disease progression is needed.

### Modulation of intracellular signalling in response to PXDN dysregulation

Kinase signalling plays a pivotal role in cancer progression. Dysregulation of extracellular cues, such as aberrant matrix cross-linking, impact significantly on downstream intracellular signalling networks [74]. Whilst investigation into the role of PXDN on intracellular signalling in cancer is limited, research in non-transformed cells has implicated PXDN in the regulation of key signalling networks. For example, endothelia cells transfected with PXDN siRNA show reduced migration, proliferation and survival *in vitro* [75] mediated through decreased phosphorylation in ERK/Akt/FAK signalling. Exogenous addition of active forms of purified recombinant PXDN (rPXDN) to TeloHAEC cardiac epithelial cells increased phosphorylation of Akt, ERK1/2 and FAK, whilst addition of a catalytically inactive form of PXDN had no effect [13].

PXDN may also play a role in modulating PI3K/Akt signalling in cancer (Figure 4). In U87.MGΔEGFR glioblastoma cells with constitutive expression of Epidermal growth factor receptor (EGFR) — a known regulator of PI3K/Akt signalling — PXDN was the most highly up-regulated gene [44]. siRNA knockdown of PXDN in HEY ovarian cancer cells also reduced phosphorylation of PI3K/Akt, leading to reduced invasion and migration [28]. However, whether this reduction in cell invasiveness was the result of changes in PI3K/Akt signalling was not examined. On the other hand, breast cancer cells with siRNA knockdown of PXDN showed decreased proliferation whilst maintaining Akt and ERK phosphorylation [76].

Other signalling pathways that have been shown to be modulated in endothelial cells by rPXDN protein include PDGFb, HB-EGF, CXCL, Hey-1, ID-2, SNAI-1 [13], FAK and ERK1/2 [75]. PXDN depletion has also been shown to reduce ERK signalling in hypoxia treated pulmonary artery smooth muscle cells [77] and attenuated a hydrogen peroxide induced increase in ERK1/2 phosphorylation in vascular smooth muscle cells [78]. Further studies are required however to determine the effects of PXDN expression in the solid tumour setting where many of these signalling pathways are also dysregulated.

## Considerations for therapeutic targeting of PXDN

### Effects of PXDN expression levels on current cancer treatments

There is evidence to suggest that PXDN may influence tumour sensitivity to therapies. The IC_50_’s of several kinase inhibitors were all reported to be higher in cell lines with high PXDN expression compared with low PXDN. However these results were not separated for different cancer types [22]. Additionally, in the PyMT mouse model of breast cancer, treatment with paclitaxel or doxorubicin resulted in remodelling of the ECM which included an observed increase in collagen IV abundance [79]. Whilst cross-linking was not measured, this increase in collagen IV presence appeared to induce invasion of PyMT cells, and invasion could be attenuated upon the administration of clinical FAK inhibitors [79]. Given that PXDN depletion using siRNA reduced FAK phosphorylation in HUVEC cells, it could be speculated that PXDN expression and/or activity, and FAK signalling may work together to play a role in the invasion of breast cancer cells, although the direct interplay between FAK inhibitors and PXDN expression and activity should be further explored in this setting.

### Possible future therapeutics targeting PXDN in cancer

The increased expression of PXDN in many cancers, as well as its links to increased cell proliferation, migration, invasion, and metastasis, suggest that it may be a viable candidate for therapeutic targeting in cancer. At present, no PXDN specific inhibitors have been developed. There are, however, a number of inhibitors that target peroxidases more broadly which have been shown to inhibit PXDN activity, including 4-aminobenzoic acid hydrazide (4-ABAH) [5,7,80], phloroglucinol (PHG) [4,5,9,59,75,81,82], methimazole [9,83] and 3-aminotriazole [9,83]. Additionally, collagen IV cross-linking is partially inhibited by urate [58,59], iodide [5,9] and thiocyanate [5].

A key consideration will be the broad inhibition of multiple members of the peroxidase family including MPO and EPO, both of which are known to play important roles in solid tumours, contributing to tumour growth, collagen deposition and metastasis [84]. Deficiencies in MPO and EPO are not known to lead to negative health effects [85,86], however both MPO and EPO have been shown to be internalised by fibroblasts, increasing their collagen deposition and migration [87], and endothelial cells resulting in increased proliferation, invasion and angiogenesis [12]. In this context, inhibitors targeting MPO, EPO and PXDN may have favourable outcomes for treating cancer compared with targeting single peroxidase enzymes alone.

Given that PXDN mediated cross-linking of collagen IV is an essential process in basement membrane generation and repair, inhibition of this process outside of the tumour microenvironment may have negative effects. PXDN does not appear to be essential in development since mice with homozygous deletion of PXDN present with eye defects but no other severe morphological changes [88]. However, the depletion of PXDN in the context of wound healing should be carefully considered. Targeting of peroxidase inhibitors specifically to the tumour microenvironment may be able to reduce the impact of these potential undesirable effects.

An alternative approach would be to target post-translational regulators of PXDN. This approach has been trialled *in vitro* with inhibitors such as CMK [63], decanoyl-RVKR-chloromethyl ketone and a-1 antitrypsin Portland variant [64], which target Furin and other pro-protein convertases, as well as by utilising methionine as a hypohalous acid scavenger [80]. Each of these approaches will likely exhibit pleiotropic effects on tissue homeostasis and disease, and so the potential impact of off-target effects should be carefully considered.

Ultimately, more research is needed to understanding the exact role that PXDN plays in different cancer types and how this is regulated before researchers and pharmaceutical companies consider PXDN to be an economically viable target for the development of targeted therapies.

## Perspectives

- Peroxidasin is an enzyme with an emerging role in tumour progression and metastasis that is involved in extracellular matrix (ECM) remodelling and modulating the composition and organisation of the ECM.
- Peroxidasin promotes cancer cell invasion, angiogenesis, and has been associated with increased metastasis.
- Further research is required to better understand the molecular mechanisms of peroxidasin in cancer progression, as well as downstream signalling pathways and interacting partners of peroxidasin. This will allow for the development of targeted therapies for cancer treatment.

## Abbreviations

ECM: extracellular matrix
EGFR: epidermal growth factor receptor
EMT: epithelial to mesenchymal transition
HOBr: hypobromous acid
HOCl: hypochlorous acid
LLR: leucine rich repeat
LPO: lactoperoxidase
MPO: myeloperoxidase
PXDN: peroxidasin
ROS: reactive oxygen species
VWC: von Willebrand factor type C
